# Refining the Loop of Continuous Improvement

**DOI:** 10.1093/geroni/igab046.158

**Published:** 2021-12-17

**Authors:** Robert Maiden, Jan Abushakrah

**Affiliations:** 1 Alfred University, Alfred, New York, United States; 2 Portland Community College, Portland, Oregon, United States

## Abstract

Academic assessment of student competency is essential to measure learning within a gerontology program. In its self-evaluation, a program must assess its student learning outcomes. JoAnn Damron-Rodriguez et al. (2019, p. 423) proposed a systematic approach that has several levels. The key is to utilize a competency-based education model. Moreover, to satisfy workforce goals the gerontology program must adopt the AGHE competencies that reflect the knowledge, skills, and attitudes necessary to serve older adults at an acceptable level of care. The next step involves generating well-articulated quantitative or qualitative measures of student learning outcomes (SLOs) consistent with the program’s mission statement that include twelve competency domains. SLO measures include test grades, assignments, projects, portfolios, field experiences, essay questions, multiple choice items, and so on. The program’s enhancement loop requires the evaluation of SLOs, faculty discussion of them, and a continuous modification cycle "closing the loop" to reach the program’s goals.

